# Overcoming Therapy Resistance and Relapse in TNBC: Emerging Technologies to Target Breast Cancer-Associated Fibroblasts

**DOI:** 10.3390/biomedicines9121921

**Published:** 2021-12-15

**Authors:** Farhana Mollah, Pegah Varamini

**Affiliations:** 1 Faculty of Medicine and Health, School of Pharmacy, University of Sydney, Sydney, NSW 2006, Australia; fmol0456@uni.sydney.edu.au; 2 Sydney Nano Institute, University of Sydney, Sydney, NSW 2006, Australia

**Keywords:** triple-negative breast cancer, breast cancer-associated fibroblasts, breast cancer, cancer-associated fibroblasts, chemoresistance, nanotechnology, microRNA, antibodies

## Abstract

Breast cancer is the most diagnosed cancer and is the leading cause of cancer mortality in women. Triple-negative breast cancer (TNBC) is an aggressive form of breast cancer. Often, TNBC is not effectively treated due to the lack of specificity of conventional therapies and results in relapse and metastasis. Breast cancer-associated fibroblasts (BCAFs) are the predominant cells that reside in the tumor microenvironment (TME) and regulate tumorigenesis, progression and metastasis, and therapy resistance. BCAFs secrete a wide range of factors, including growth factors, chemokines, and cytokines, some of which have been proved to lead to a poor prognosis and clinical outcomes. This TME component has been emerging as a promising target due to its crucial role in cancer progression and chemotherapy resistance. A number of therapeutic candidates are designed to effectively target BCAFs with a focus on their tumor-promoting properties and tumor immune response. This review explores various agents targeting BCAFs in TNBC, including small molecules, nucleic acid-based agents, antibodies, proteins, and finally, nanoparticles.

## 1. Introduction

Breast cancer is the most common cancer in women worldwide [1,2]. According to the WHO, 2.3 million women worldwide were diagnosed with breast cancer in 2020, with 685,000 deaths [3]. Around 15–20% of breast cancer is triple-negative breast cancer (TNBC), the most aggressive subtype that accounts for 25% of all breast cancer deaths [4,5]. TNBC is characterized by the absent expression of cellular receptors, estrogen, progesterone, and human epidermal growth factor receptor 2 (HER2) receptors [6]. Due to the lack of these receptors, estrogen, progesterone hormonal, and HER2 targeted therapies are ineffective against TNBC. Therefore, nonspecific chemotherapy is the predominant treatment form [7], which is associated with severe side effects, including, but not limited to, skeletal muscle damage, cardiotoxicity, and myelosuppression [8,9]. Reduced treatment dose is often necessary to limit side effects generated by untargeted chemotherapeutic agents, but often leads to poor treatment outcomes and cancer recurrence [9]. Frequently, TNBC relapse progresses to a metastatic stage with a poor 5-year survival rate of 12% [10,11]. 

### 1.1. The Tumor Microenvironment

The tumor microenvironment (TME) plays a central role in resistance to therapy and tumor progression in cancer [12]. The cell and acellular components surrounding malignant cells are recruited to form a TME through communication factors, such as chemokines and cytokines [5]. Various components shape the TME, including cancer-associated fibroblasts (CAFs), endothelial cells, adipocytes, mesenchymal stem cells (MSCs), macrophages, vessels, pericytes, and the extracellular matrix (ECM) [5,13,14]. The crosstalk between cancerous cells and TME enables tumor progression and transformation from in situ to an invasive phenotype [12,15,16]. Dynamic interactions between the components of TME and cancer cells affect tumorigenesis and therapeutic resistance through various mechanisms, such as signal transduction, paracrine signaling, and autophagy [13,14,17,18]. Additionally, the TME contributes significantly to the epithelial-to-mesenchymal transition (EMT). This transition involves various processes to replace the epithelial properties of cancer cells with the mesenchymal characteristics that allow the invasion of adjacent tissues [19]. Furthermore, EMT provides cancerous cells with the ability to metastasize in other organs, forming secondary tumor sites, where tumorigenesis is again dependent on the microenvironment according to the ‘seed and soil’ hypothesis [19,20]. Therefore, the components of TME and crosstalk with cancer cells are crucial to tumorigenesis and metastasis. 

The most abundant types of cells in the TME are CAFs, which are activated fibroblasts [21]. CAF activation occurs through epigenetic modification, as is frequently reported to be through the promoter DNA methylation or post-transcriptional modification [22]. CAFs are defined with their spindle-shaped morphology and mesenchymal features, such as the overexpression of alpha-smooth muscle actin (α-SMA), fibroblast activation protein (FAP), vimentin (VIM), and platelet-derived growth factor-alpha (PDFG-α) [23,24]. They lack the characteristics of non-mesenchymal cells (endothelial and epithelial) and the genetic mutations found in cancerous cells [23,25]. α-SMA was expressed in CAFs and was associated with lymph node metastasis and a poor prognosis [26,27,28]. FAP is a cell surface glycoprotein located on CAFs, which enzymatically remodels ECM and causes cellular migration [29]. FAP is selectively expressed in the tumor microenvironment and is undetectable in healthy tissue [30]. Other identifiers of pro-tumorigenic CAFs in breast cancer include a zinc-dependent metalloproteinase, CD10, and the G protein-coupled receptor 77 (GPR77) [31]. GPR77 promotes chemoresistance, expresses vascular endothelial growth factor (VEGF), and induces angiogenesis in breast TME through insulin-like growth factor 1 (IGF1) signaling [31,32]. Paracrine PDFG-AA/-BB signaling in breast cancer distinguishes its TME by causing desmoplasia and tumorigenesis [33]. Desmoplasia is characterized by a collagenous microenvironment due to the presence of CAFs, which is associated with invasiveness and a poor prognosis in breast cancer [34,35].

### 1.2. Breast Cancer-Associated Fibroblasts

**Figure 1:**
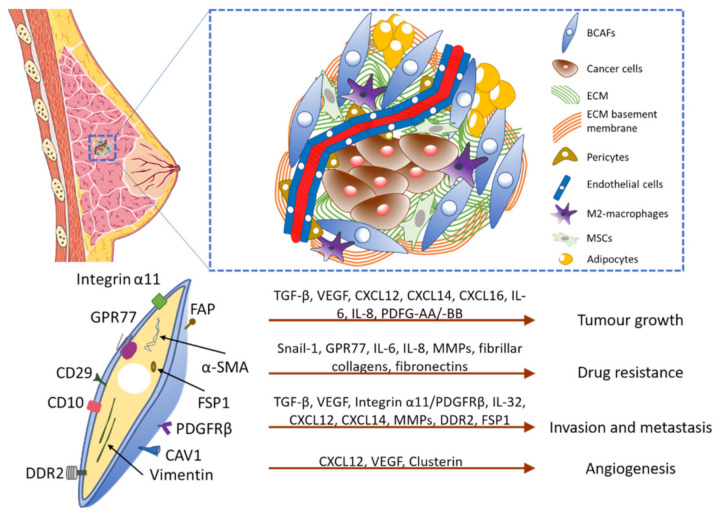
Breast cancer tumor microenvironment, BCAF biomarkers, and pro-tumorigenic functions. The breast cancer tumor microenvironment possesses BCAFs, ECM components and various other cell types, including endothelial cells, M2-macrophages, mesenchymal stem cells (MSCs), adipocytes, and pericytes. BCAFs actively contribute to the production of ECM components and promote M2-macrophages recruitment and metastasis. In the bottom half, biomarkers of BCAFs and the mechanisms of pro-tumorigenic functions are displayed. Some of the biomarkers are Integrinα11, GPR77, CD10, DDR2, α-SMA, Vimentin, FSP1, CD29, CAV1, FAP, and PDGFRβ. BCAFs contribute to tumor growth, drug resistance, invasion, and angiogenesis by secreting various chemokines (CXCL16, CXCL14 and CXCL12), growth factors (TGF-β and VEGF), interleukins (IL-6, IL-8, and IL-32), ECM (fibrillar collagens and fibronectins) and possessing other factors/receptors, such as MMPs, Snail-1, GPR77, Integrin α11/PDGFRβ, DDR2, and clusterin.

Breast cancer-associated fibroblasts (BCAFs) are the CAFs present in breast cancers with genetic and phenotypic characteristics similar to CAFs [28]. CAFs originate from a diverse range of cells, including endothelial cells [36], adipocytes [34], pericytes [37], and MSCs [38]. Although CAFs were derived from endothelial cells and pericytes, the derivation was not tested in breast cancer models similar to other cancers [36,37]. BCAFs have also been derived from adipocytes that lead to a desmoplastic microenvironment [34]. BCAFs can originate from MSCs, which contribute to angiogenesis through up-regulation of clusterin leading to tumorigenesis [38]. BCAFs possess the fibrillar collagen receptor, DDR2, which rearranges collagen fibers to develop an invasive and metastatic TME [39]. Additionally, integrin α11 in BCAFs interacts with platelet-derived growth factor receptor beta (PDGFRβ) and promotes invasiveness by activating c-Jun N-terminal kinase (JNK) and producing a matricellular protein, tenascin C (Figure 1) [40].

BCAFs have heterogenous subpopulations in TNBC [41,42] and these different subpopulations are identified by various markers, including CD29, FAP, αSMA, PDGFRβ, FSP1, and caveolin 1 (CAV1) [41]. CAV1, CD29/integrin β1 and fibroblast-specific protein 1 (FSP1)/S100-A4 have been used as markers of BCAFs [41]. However, previous studies showed conflicting results for the impact of CAV1. One study demonstrated that CAV1 promotes invasion [43] and the other reported loss of CAV1 led to invasion [44]. Presence of FSP1 is correlated with poor prognosis and metastasis in breast cancer [45,46]. Two of the subpopulations of BCAFs found in TNBC were termed S1 and S4. S1 has low CAV1, medium CD29 and high FAP, α-SMA, PDGFRβ, FSP1 expressions. On the other hand, S4 is characterized by the presence of high levels of CD29 and α-SMA, medium levels of PDGFRβ and FSP1, low-to-negative expression of CAV1 and negative for FAP. S1 and S4 also differ in their function of developing an immunosuppressive environment such as the S1-enriched TNBC has higher FOXP3+ T cells and lower CD8+ T lymphocytes expression, compared to the S4 containing TNBC [41].

Additionally, one study revealed six subpopulations of TNBC tumor-derived BCAFs with unique gene expressions [42]. They were described as α-SMA^high^, lymphocyte antigen 6 complex, locus C1 (LY6C1^high^), leukocyte surface antigen CD53^high^, Cellular Retinoic Acid Binding Protein 1 (CRABP1^high^), CD74^high^ and cyclin CAFs (which expressed cyclin-dependent kinase 1) due to the high level of expression of those genes. Furthermore, the study compared the TNBC BCAFs with pancreatic tumors. This comparison revealed that only three biomarkers are common between the two cancers, CD74^high^, α-SMA^high^ and LY6C1^high^ [42]. These data illustrate the complex heterogeneity of BCAFs in TNBC and that it can be different from other types of cancers.

Furthermore, BCAFs can promote TNBC chemoresistance by supporting metabolic adaptations of TNBC [47]. The interaction between BCAFs and TNBC is explained by the reverse Warburg effect, when TNBC cells are metabolically supported via glycolysis. Lactate is produced by BCAFs though aerobic glycolysis and is transported to tumor cells to power tricarboxylic acid (TCA) cycle. TNBC cells are more dependent upon glucose metabolism compared to other breast cancer subtypes, such as ER positive, PR positive, and HER2 positive, which have been explained by the overabundance of the glucose transporter protein MCT4 in TNBC cells [48,49]. Furthermore, higher levels of glycolysis and lactate permutation were observed in chemoresistant TNBC cells [50]. Through these mechanisms, BCAFs assist TNBC cells with glucose metabolism and enable chemoresistance.

BCAFs are involved in the progression, invasion, and metastasis of breast cancer, as well as chemotherapeutic resistance (Figure 1) [28,51]. BCAF secretes a variety of growth factors and cytokines, such as the transforming growth factor β (TGF-β), VEGF, and the C-X-C motif chemokine ligand 12 (CXCL12) that promotes tumor growth, metastasis, and invasion [52,53,54]. BCAF secretes CXC-chemokine ligands 14 and 16 (CXCL14 and CXCL16). CXCL14 is correlated with a poor prognosis of breast cancer and contributes to EMT, invasiveness, and metastasis using the atypical G protein-coupled receptor, ACKR2 [55]. CXCL16 is strongly associated with phosphodiesterase 5 (PDE5) levels and poor clinical outcomes [56]. Furthermore, BCAF secreted cytokines, such as interleukin 6 (IL-6) and IL-8, which enhance tumorigenesis and metastasis [57]. Another BCAF cytokine, IL-32, facilitates crosstalk between breast cancer cells and BCAFs through its interaction with the integrin β3, and promotes tumor invasion and metastasis [58]. 

Furthermore, BCAFs produce extracellular matrix (ECM) components, such as fibrillar collagens and fibronectins, in order to remodel the ECM and form physical barriers against drug penetration [30,59]. BCAF generated type I collagen, which was shown to reduce chemotherapeutic drug uptake and decrease sensitivity to therapy [60]. BCAF-induced chemoresistance was also demonstrated to be due to fibronectin and other stromal factors secreted by BCAF, such as the IL-6 and matrix metalloproteinases (MMP) [21,61]. BCAFs express high levels of the transcription factor, Snail-1, alongside cancer cells, which decrease chemotherapeutic sensitivity and enable EMT [62]. Since BCAFs have crucial functions in TNBC therapy resistance and metastasis, targeting BCAFs is a promising strategy to overcome TNBC disease burden.

This review discusses various therapeutic agents under investigation that target BCAFs in TNBC, highlighting the importance of these components in TME for the future development of novel targeted therapeutics against TNBC. Additionally, it will provide information on the opportunities and challenges presented by BCAF-targeted therapeutic strategies for the unmet challenge of aggressive TNBC through their prominent role in the tumor microenvironment.

## 2. Therapeutic Strategies Targeting BCAFs

Given the crucial role of BCAFs in the progression of breast cancer, many studies have been conducted to target them using various strategies, such as nanotechnology platforms and small molecules (Table 1) [26,30]. As each strategy employs a unique mechanism to target BCAFs, it is important to categorize them in the following sections.

### 2.1. Free Small Molecules

In the following section, several small molecules that are approved for application in various diseases but are repurposed to be used as potential anti-BCAF candidates are discussed. The advantage of repurposing drugs for the targeting of BCAFs is that their clinical translation will be faster than that of novel compounds, which will require further investigation for clinical translation and approval.

#### 2.1.1. TGF-β Inhibitors

TGF-β1 is a cytokine that binds to ligand binding receptors and recruited the receptors termed TGF-βRII and TGF-βRI, respectively [83,84]. TGF-β1′s engagement with TGF-βRII causes the recruitment and activation of the TGF-βRI, which phosphorylates the SMAD proteins (SMAD2 and 3). This pathway regulates the ECM genes to express ECM components, such as collagens and fibronectins, which produce dense fibrotic tissue [85]. 

Synthesized artesunate (ARS) and dihydroartemisinin (DHA) are derivatives of artemisinin extracted from Sweet Wormwood (*qinghao*), which have previously demonstrated antitumor activity in leukemia, colon cancer, fibrosarcoma, and breast cancer [86]. ARS and DHA negatively impacted some BCAFs in an orthotopic 4T1 model of mice by suppressing TGF-β signaling [63]. In this study, the first BCAFs were isolated from murine models of MMTV-PyMT to represent a subset of TNBC patients with luminal androgen receptor expression [87]. The BCAFs were then treated with ARS and DHA. Although there was no significant impact on cell viability compared to the control, the expression of CAF markers, including α-SMA, FAP and fibronectin, was significantly reduced (*p* < 0.01). Additionally, ARS and DHA were shown to repress the TGF-β signaling to inhibit BCAF activation and reduce tumor growth and metastasis in vivo [63]. Significantly decreased TGF-β1 and phosphorylated SMAD3 levels showed that ARS and DHA were inhibiting the TGF-β signaling. 

Pirfenidone is a TGF-β antagonist and has been approved for clinical use to treat idiopathic pulmonary fibrosis [88]. It has been effective as an antifibrotic agent in various preclinical studies with different conditions, such as nonalcoholic steatohepatitis and pancreatic cancer [89,90]. Takai et al. used pirfenidone to target BCAFs derived from syngeneic and xenograft models of TNBC [54]. Pirfenidone inhibited BCAF proliferation and fibrosis. It also caused apoptosis of both cancer cells and BCAFs. Furthermore, this group showed that pirfenidone inhibited fibrosis and TGF-β signaling but did not prevent the growth of TNBC tumors in vivo. The combination of pirfenidone with doxorubicin synergistically inhibited tumor growth and metastasis in the 4T1 syngeneic tumor model of TNBC. The strength of this study is that they isolated BCAFs from breast cancer patients and characterized them using Vim, FAP and the absence of an epithelial tumor marker, pan-cytokeratin [54]. One limitation of this study was that CAFs were injected along with cancer cells in mice, so the impact on the basal level of BCAFs in breast TME was not possible to be determined. Another limitation is that they did not explore the subpopulation of BCAFs that was impacted by the treatment.

In a different study, pirfenidone reduced fibronectin levels, hyaluronan area, and mechanical force in 4T1 tumors. It also increased perfusion in ECM, which improved doxorubicin efficacy when used in combination with pirfenidone. In this study, levels of α-SMA remained consistent after pirfenidone treatment. The authors concluded that the BCAFs were not affected in their TNBC mice model [64]. However, this could have been due to various other contributing factors to determine the presence and variations in BCAF levels that were not taken into consideration in this conclusion by the authors. For example, another detection method of BCAFs are the fibronectins they produce. Given that the level of fibronectins was reduced in this study after treatment, a subset of BCAFs is more likely to be affected by pirfenidone. In another study by Aboulkheyr Es et al., pirfenidone was tested against BCAFs co-cultured with the TNBC cell line, MDA-MB-231, in a microfluidic device [65]. In this work, pirfenidone significantly inhibited TNF-β secretion, PD-L1 expression, and BCAF migration [65]. Taken together, there is substantial evidence that pirfenidone has an inhibitory effect on BCAFs’ function and migration [54,65]. However, further research is required to establish that pirfenidone can target different subsets of BCAFs and the basal level of TNBC BCAFs in vivo.

Tranilast is an antihistamine drug and TGF-β inhibitor. This drug was shown to effectively target BCAFs in TNBC mice models [66]. Tranilast decreased ECM components and increased perfusion and infiltration of T cells. When combined with Doxil^®^ (liposomal doxorubicin) to treat TNBC, it improved treatment efficacy, expression of immunostimulatory macrophage M1, and enhanced immune checkpoint blocking antibodies [66]. Another novel strategy used emodin (6-methyl-1,3,8-trihydroxyanthraquinone), which has demonstrated anti-inflammatory, antiviral, anticancer, and pro-apoptotic activities [91]. Hsu et al. extracted BCAF from tumor tissues of TNBC patients and examined the effects of BCAF conditioned medium on epithelial BT-20 breast cancer cells [67]. Emodin inhibited cell migration and EMT through TGF-β induced by BCAFs [67].

#### 2.1.2. Dual Targeting Agents: Combined Anti-BCAF and Other Pharmacological Activity

Several anticancer agents have been found to have an impact on BCAFs. In one study, the BCAF-inhibitory potential of 138 compounds was estimated using the Cancer Genome Atlas and Genomics of Drug Sensitivity in Cancer databases of TNBC patients and associations with α-SMA expression. BCAFs have different expression levels of α-SMA (high and low) in different tumor models [68]. Embelin is a quinone derived from *Embelia ribes Burm* plants and one of the 24 agents that were estimated to have an impact on α-SMA levels [68,92]. Embelin has shown anticancer activity in a variety of cancers, such as oral squamous cell carcinoma and lung cancer [92]. Embelin was tested in two α-SMA classified tumor types, 4T1 (high α-SMA) and 4T07 tumor models (low α-SMA) [68]. Embelin’s reduction of tumor volume was higher in 4T1 tumor (high α-SMA) than in 4T07 tumor (low α-SMA) [68]. It is likely that there are two populations of BCAF, however, further characterization of these populations is required.

Cisplatin is a DNA crosslinker, which is traditionally used for the treatment of testicular, ovarian, bladder, head and neck, lung, and breast cancer [93]. In a clinical trial, short-term cisplatin treatment was used as part of a combination therapeutic strategy with nivolumab to treat metastatic TNBC. Cisplatin caused up-regulation of immunogenic genes and increased the response rate to PD-1 blockade by altering the TME [94]. Balog et al. investigated the immunophenotype of the TNBC 4T1 mice model, as well as the tumor stroma following treatment with cisplatin. They observed that FAP proteolytic activity decreased as a result of cisplatin treatment [69]. It was speculated that the anticancer activity of cisplatin involved BCAFs inactivation [69]. However, further investigation is required to validate the study results, such as determining the expression of other BCAF markers, such as α-SMA and collagen.

BCAF secreted chemokine, CXCL12, binds to the CXCR4 chemokine receptor in cancer cells and regulates signaling pathways that allow growth, chemotherapy resistance, and metastasis [95,96,97]. Bicyclam AMD3100 is a CXCR4 antagonist and an approved drug for hematopoietic stem cells mobilization. It enables stem cell transplantation and is used for hematologic malignancy and other diseases, such as bone marrow failure and sickle cell disease [95]. In cocultures of TNBC cells and BCAFs (both 2D and 3D), AMD3100 normalized cancer cell growth to the level observed in cells without any CXCL12 signaling [53]. Furthermore, CXCL12 and CXCR4 signaling stimulated cell growth and invasion, which was prevented by AMD3100 [53]. 

Cabozantinib is an inhibitor of the tyrosine kinase receptor MET, which functions as an anticancer agent [98]. Hepatocyte growth factor (HGF) is secreted from CAFs at elevated levels and stimulates MET signaling [70]. Cabozantinib prevented invasion and growth of HGF-overexpressed TNBC cells (MET positive MDA-MB-231 and HCC70) co-cultured with BCAFs, which also had HGF overexpression. This treatment did not affect MET negative TNBC cell lines, which reflected the specificity of this inhibitor [70]. However, this strategy can only be used against certain subtypes of TNBC where MET overexpression exists and a subset of BCAFs that has HGF expression.

A recent study developed a library of pyrrolo[2,3-d]pyrimidine-based compounds, including 15d (7-(4-((7-(2-Fluorobenzyl)-7H-pyrrolo[2,3-d]pyrimidin-2-yl)-amino)-1H-pyrazol-1-yl)-N-hydroxyheptanamide) and 15h (7-(4-((7-(3-Fluorobenzyl)-7H-pyrrolo[2,3-d]pyrimidin-2-yl)- amino)-1H-pyrazol-1-yl)-N-hydroxyheptanamide) [71]. They inhibited the histone deacetylase (HDAC) and Janus kinase (JAK) isoforms. These compounds were rationally created as dual targeting agents because the efficacy of HDAC inhibitors is limited due to the activation of the leukemia inhibitory factor receptor (LIFR) and JAK. A total of 15 d and 15 h abrogated LIFR-JAK signaling generated by CAFs, but only 15 h was also effective in diminishing tumor growth in the TNBC (MDA-MB-231) xenograft [71].

The Notch signaling pathway regulates cell–cell communication. Overexpression of Notch receptors is highly associated with the aggressive phenotype of TNBC, including invasiveness and resistance to chemotherapeutics [99]. Notch receptors can be blocked by a γ-secretase inhibitor, DAPT (N-[N-(3,5-difluorophenacetyl)-lalanyl]-S-phenylglycine t-butyl ester), which has shown activity against TNBC [99,100]. DAPT led to a reduced invasion of MDA-MB-231 co-cultured with CAFs [72]. It also inhibited CXCL8 (a pro-metastatic chemokine) in TNFα stimulated co-cultures of MDA-MB-231 and CAFs. The interaction between TNBC and CAFs amid the presence of TNFα resulted in increased migration and invasion. This was prevented by DAPT treatment through the inhibition of Notch signaling [72].

### 2.2. Nucleic Acid-Based Targeting Agents

MicroRNAs (miRNAs) are non-coding RNAs that regulate 30% of the gene expression of mRNA by binding to specific sites [101]. MiRNAs are aberrantly expressed in breast cancers compared to healthy breast tissue [102]. Significant deregulation of microRNA-21 (miR-21), miR-155, miR-145, and miR-125b has been discovered in breast cancers [102]. The overexpression of these miRNAs plays a crucial role in tumor progression and invasion in breast cancer [102]. Various miRNAs mimic and antisense oligonucleotides have been utilized to correct the expression of miRNA in BCAFs [103] as discussed in this section (Table 1).

#### 2.2.1. Small Molecules Targeting miRNAs

MiR-21 is abundantly expressed in breast cancers and is known to be associated with advanced disease and poor survival [104,105,106]. The Kang group confirmed that miR-21 is up-regulated in in vitro and orthotopic TNBC (MDA-MB-231) mice models [73]. This group corrected miR-21 expression using AC1MMYR2 (AMR), a miR-21 small molecule inhibitor, which also reduced tumor growth by reversing the transition of epithelial to mesenchymal cells [73]. They later demonstrated that miR-21 up-regulation was strongly associated with breast cancer lymph node metastasis. They also found that miR-21 activation is directly related to the metastatic effect of BCAFs through NF-κB axis [107]. Treatment with AMR decreased levels of α-SMA and FAP-α. Combination therapy using AMR and paclitaxel in an in vivo model reduced tumor growth and lung metastasis. This study shows that AMR effectively targets both α-SMA and FAP-α expressing BCAFs and TNBC depending on the presence of miR-21 [107].

Curcumin is the active compound in the *Curcuma longa* herb, which is more commonly known as turmeric [108]. It has been studied in various clinical trials due to its anticancer activities and high safety (up to 12 g/day) for human consumption [109,110]. The Aboussekhra laboratory determined that curcumin treatment upregulated p16 in BCAFs, which caused inactivation and senescence (growth arrest) of BCAFs [75]. Furthermore, curcumin inhibited the migration and invasion capabilities of MDA-MB-231 cultured with BCAF-conditioned medium and reduced BCAF secretion of SDF-1, IL-6, MMP-2, MMP-9, and TGF-β [75]. In a later study, the Aboussekhra group revealed that the up-regulation of p16 induced the miR-146b-5p expression, which led to reduced level of BCAF-secreted factors and prevented EMT, migration, and invasion of MDA-MB-231 [76].

#### 2.2.2. Mimics and Anti-miRNAs

The TGF-β pseudoreceptor, bone morphogenetic protein and activin membrane-bound inhibitor (BAMBI) are mimics of the TGF-β receptor without serine/threonine kinase domain that is necessary for TGF-β/SMAD signaling [111]. The transduction of cells with BAMBI using the lentiviral vector inhibited TGF-β/SMAD signaling [77]. The transformation of bone marrow mesenchymal stem cells (BM-MSCs) into CAFs has been demonstrated to occur in the presence of TGF-β and cancerous cells. BAMBI transduced mesenchymal cells that were co-inoculated with MDA-MB-231 cells and caused the inhibition of tumor growth and metastasis, while the parent mesenchymal cells promoted tumor growth and metastasis. Furthermore, BAMBI treatment prevented BCAFs differentiation from BM-MSCs [77].

The down-regulation of a tumor suppressor miRNA, called let-7b, caused the activation of BCAFs [78]. In this study, BCAF activation was demonstrated by the presence of α-SMA, SDF-1 and TGF-β1. The BCAF activation occurred through IL-6 induction, which led to a high level of IL-8 expression, which subsequently promoted the EMT in cancer cells. Additionally, deregulation of let-7b was shown to enhance the migration and invasion capacity of BCAFs and tumor growth. Let-7b mimics restored the levels of let-7b in BCAFs and reduced the pro-tumorigenic functions of BCAFs. Furthermore, BCAF inactivation was confirmed by demonstrating decreased levels of α-SMA, IL-8 and TGF-β1 as well as CXCL12 (also known as SDF-1). Let-7b mimics inhibited migration and invasion in TNBC cells (MDA-MB-231) in vitro [78]. Further studies are required to establish that let-7b mimics are effective against different sub-groups of BCAFs and in TNBC in vivo models.

Exosomes are vesicles of approximately 40 to 100 nm in size and are used as intercellular communication mediators in cancer [112]. Anti-miRs were utilized to inhibit the function of exosomes secreted by BCAFs [79]. These exosomes overexpressed miR-21, miR-143, and miR-378e, which increased stemness, EMT phenotype, and proliferation in TNBC. MDA-MB-231 cancer cells were transfected with anti-miR-21, -143, and -378e, which inhibited the effects of BCAFs’ exosomes [79]. However, the group only investigated the effect of anti-miRs in a transfected breast cancer cell line (T-47D) in vitro. The efficacy of anti-miRs needs to be confirmed in TNBC models in the future.

#### 2.2.3. Aptamers

Aptamers are single-stranded DNA and RNA oligonucleotides that can be designed into structures that target small molecules, proteins, and live cells [113]. These aptamers are generally produced through the systematic evolution of ligands through the exponential enrichment process (SELEX), where the oligonucleotides are projected to iterative partitioning to form the desired structure [114,115].

Locked nucleic acid (LNA)-i-miR-221 oligonucleotide treatment significantly inhibited BCAF and TNBC (MDA-MB-231) cell growth and migration [80]. LNA-i-miR-221 prevented the function of miR-221 that promotes cell growth and migration. The mechanism of action of LNA-i-miR-221 was revealed to be through down-regulation of the expression of the A20 ubiquitin editing enzyme and increased transcription factor c-Rel and connective tissue growth factor. The BCAFs used in this study were derived from mammary ductal carcinoma mastectomies (the TNBC status of the mastectomies was not confirmed) [80]. Further studies should investigate the efficacy of LNA-i-miR-221 in a coculture of TNBC cancer and BCAF cells and in in vivo models of TNBC that would more accurately demonstrate the efficacy of the agent in tumor growth and progression.

MiR-9 has been proven to be highly up-regulated in primary TNBC BCAFs compared to normal fibroblasts [81]. MiR-9 transformed normal fibroblasts into CAFs, which then promoted migration, invasion, and motility. This was effectively inhibited using locked nucleic acid targeted against miR-9 (LNA-9), which reversed the migration, invasion, and motility phenotype in BCAF. It was speculated that TNBC cells and BCAFs secrete exosomes containing miR-9, which increase the motility of TNBC cells and BCAFs [81]. Their study design consisted of using immortalized normal fibroblasts transfected with miR-9 to represent CAFs and injecting them alongside TNBC cells (MDA-MB-468) into SCID mice to demonstrate BCAF-promoted tumorigenesis [81]. This could have been improved by using TNBC BCAFs because it could mimic the functions of BCAFs more closely compared to immortalized normal fibroblasts transfected with miR-9. Furthermore, the sub-populations impacted by this treatment should also be determined.

BM-MSCs have the ability to differentiate into other cell types, such as CAFs and tumor-associated macrophages [116]. The platelet-derived growth factor receptor (PDGFRβ) has a significant role in MSC recruitment to the tumor site [117]. Furthermore, the basic fibroblast growth factor and platelet-derived growth factor BB support the function of bone marrow in the dissemination of breast cancer cells [118]. The nuclease resistant aptamer called Gint4.T is an RNA-based oligonucleotide that specifically binds with high affinity to PDGFRβ and blocks its activity [119]. Cocultures of BM-MSC and TNBC cells resulted in increased mRNA levels of α-SMA, FAP and FSP-1, which confirmed that BM-MSC can transdifferentiate into CAF-like cells [82]. The Gint4.T inhibited BCAF formation from BM-MSC and the metastatic ability of the BM-MSC-induced TNBC (MDA-MB-231) tumor in vivo [82].

Although this approach may be potentially promising, the regulation of miRNAs is complex, as miRNA may have various functions that can complicate the further development of RNA-targeting agents and their clinical translation [103]. Only one aptamer, pegaptanib (Macugen^®^), has been approved in the last 3 decades since its discovery due to challenges such as rapid excretion and low in vivo binding affinity [120,121]. Only three oligonucleotides have been in clinical trials to date; two were terminated in early phase trials and none have yet been approved for cancer treatment [122,123]. Thus, this field of research requires further preclinical validation prior to clinical translation.

### 2.3. Antibodies and Proteins

Antibodies and protein-based agents have been used to inhibit certain functions of BCAFs in TNBC [124,125]. The major concern with traditional chemotherapy drugs for TNBC is the lack of specificity and limited efficacy [126]. Therefore, as antibodies are highly specific and bind only to their target receptor, they are used in the development of targeted anti-BCAF agents and have high potential for the treatment of aggressive TNBC (Table 2).

#### 2.3.1. Antibodies

BCAFs induced chemotherapy resistance through the activation of interferons, such as Type I and II [124]. The interferon signaling of BCAFs was demonstrated by communication between interferon β1 in BCAFs and the MX1 protein in TNBC cells. Type I and II interferon signaling blocking antibodies reversed BCAF-induced chemoresistance in some TNBC cells (MDA-MB-231 and MDA-MD-157). However, this effect was not present in TNBC MDA-MB-468 cells and the different responses of TNBC cells were attributed to the TNBC subtype [124]. Herschkowitz et al. identified a molecular subtype of breast cancer cells termed as ‘claudin-low’, which was characterized by minimal expression of genes that function in epithelial cell–cell adhesion and tight junctions, such as E-cadherin and claudin 3 [140]. This claudin-low subgroup has been correlated with a poor prognosis and chemotherapy resistance [141,142]. Blocking antibodies against Type I and II interferon signaling were only effective in claudin-low cells (MDA-MB-231 and MDA-MD157) and not in the claudin-high TNBC cell line (MDA-MB-468) [124]. There is a potential for the clinical translation of this research after further investigation in animal models. This is due to the availability of clinically approved interferon receptor blockers, such as anifrolumab (a monoclonal antibody) and inhibitors of the interferon signaling pathway, such as ruxolitinib (a small molecule) [143,144].

Another novel approach involved the use of RG7386, a bispecific antibody that targets both FAP on BCAFs and the death receptor-5 in cancer cells [127]. This treatment resulted in apoptosis of cancer cells (MDA-MB-231) in vitro with or without GM05389 fibroblast coculture. RG7386 also inhibited tumor growth in a TNBC xenograft model generated by co-injection of MDA-MB-231 and fibroblasts, NIH3T3, only in the presence of FAP expressing stroma. RG7386 was effective as monotherapy and in combination with doxorubicin or irinotecan. Furthermore, RG7386 demonstrated superior anticancer activity due to the FAP target compared to conventional death receptor-5 antibodies [127]. This strategy could only be effective against FAP expressing BCAFs and further should characterize the sub-populations of BCAF against which RG7386 will be effective.

#### 2.3.2. Proteins

Integrin αvβ3, a transmembrane protein, is highly expressed in CAFs [145]. A new synthetic protein, ProAgio, was developed to target the integrin αvβ3, which caused apoptosis through the recruitment of Caspase-8 [125]. ProAgio successfully induced apoptosis in integrin αvβ3 containing TNBC BCAFs and angiogenic endothelial cells. BCAFs depletion due to ProAgio resulted in reduced intratumoral collagen, growth factors secreted by BCAF, resistance to apoptosis, and proliferation of cancer cells. Furthermore, ProAgio suppressed tumor growth and improved survival in the TNBC (MDA-MB-231) xenograft model in mice [125].

#### 2.3.3. Antibody–Drug Conjugates

The development of antibody–drug conjugates (ADCs) has been another approach to eliminating TNBC BCAFs [128]. Eribulin mesylate is a fully synthetic macrocyclic ketone that is clinically approved for metastatic breast cancer in the United States and is currently used in more than 60 countries worldwide [128,146]. ADCs are developed to address the limited specificity problem of traditional chemotherapy, where the antibody will safely deliver the drug to the target site [128]. Various eribulin-based ADCs are under investigation. Farletuzumab is a monoclonal antibody targeting the folate receptor alpha (FRA) and has been in clinical trials for the treatment of epithelial ovarian cancer [147]. FRA is abundantly expressed in TNBC and is correlated with a poor prognosis [148,149]. Furuuchi et al. developed MORAb-202 antibody–drug conjugate that is composed of eribulin mesylate and farletuzumab coupled through a cathepsin B– cleavable linker. MORAb-202 showed an FRA receptor-mediated antitumor activity and reduced the expression α-SMA of CAFs compared to the control. Furthermore, in a patient-derived xenograft TNBC model, BCAF was demonstrated to be affected by MORAb-202 through the bystander effect. This led to enhanced tumor suppression and sensitization to therapy [128].

Another approach that involves antibodies is the use of immunotoxins, which are similar to antibody–drug conjugates, except the payload is a toxin [150]. Immunotoxins are constituted of an antibody or part of an antibody that is connected to a plant or bacterial toxin [150]. When the immunotoxin binds to the target antigen in the tumor, it causes cell death. Recombinant immunotoxins, such as *Diphtheria* toxin and *Pseudomonas* exotoxin A (PE), are widely used to develop targeted immunotoxins [129,150]. Fang et al. fused PE with a specific antibody for FAP, scFv, to generate a FAP-targeted immunotoxin (αFAP-PE38) [129]. αFAP-PE38 depleted FAP positive BCAFs, changed levels of chemokines, cytokines, and MMPs, and reduced tumor growth in the 4T1 TNBC model. Combination therapy with paclitaxel further suppressed tumor growth in vivo [129].

### 2.4. Nanoparticles

Nanoparticles consist of multiple components that allow drugs or compounds to be delivered to the site of interest and unleash a certain effect [30]. They have the ability to be easily tuned and functionalized for active targeting, deliver a large quantity of therapeutics to tumor cells, and provide passive targeting via the enhanced permeability and retention (EPR) effect to reach tumor tissue (by extravasation through leaky tumor vessels). Furthermore, nanoparticles can be engineered to deliver a consistent amount of payload and release therapeutic agents with a high level of control [151]. Various nanoparticles were designed to target BCAFs in TNBC (Table 2).

#### 2.4.1. Gold Nanoparticles

Gold nanoparticles (15 nm diameter) targeting αvβ3 integrins demonstrated high efficacy in uptake and retention in MDA-MB-231 and CAFs [130]. These gold nanoparticles were functionalized with polyethylene glycol and an integrin-binding peptide, arginine-glycine-aspartate (RGD), which targets cells with αvβ3 integrin to improve tumor cell uptake [152]. The uptake of gold nanoparticles was approximately six and 12 times higher in MDA-MB-231 cells and CAFs, respectively, in comparison to fibroblasts [130]. The retention period was also 30% longer in TNBC cells and CAFs compared to fibroblasts. This delivery system could achieve optimal drug delivery to cancerous cells and CAFs [130]. In this study, CAFs were derived from melanoma tumors, and nanoparticles were tested in monocultures of CAFs and breast cancer cells. However, to better elucidate the effects of gold nanoparticles in TNBC with respect to BCAF, a model involving the coculture of BCAFs and MDA-MB-231 cells would be more beneficial and characterizing the subset of BCAFs that are impacted.

#### 2.4.2. Lipid-Based Nanoparticles

An indirect method of targeting BCAFs is by suppressing their function and blocking their secreted factors [30]. BCAFs produce tenascin C (TN-C), an extracellular glycoprotein [153,154]. Li et al. developed lipid-based nanoparticles (mixture of sulfatide and perfluorooctylbromide) nanoparticles. Sulfatide can bind to several ECM glycoproteins, including TN-C, and can be readily incorporated into the structure of lipid nanoparticles [131]. These nanoparticles were developed to selectively deliver paclitaxel to the tumor microenvironment. Paclitaxel-loaded targeted nanoparticles resulted in higher cytotoxicity and tumor inhibition than untargeted nanoparticles in the syngeneic EMT6 TNBC model of mice. This study evidenced that indirect targeting of BCAFs through TN-C can enhance the activity of chemotherapeutic agents [131].

Interleukin 10 (IL-10) that is secreted by BCAFs and is highly expressed in TNBC, contributes to immunosuppressive TME that results in immunotherapy resistance [28,155]. CXCL12 is also secreted by BCAFs and directly promotes tumor growth [96]. Another indirect BCAF targeting approach used nanoparticles loaded with IL10 and CXCL12 trap genes [132]. This nano-delivery system consisted of liposome-protamine-DNA nanoparticles, genes encoding the IL-10 or CXCL12 protein trap, and polyethylene glycol. This IL-10 trap-based nanotechnology significantly inhibited tumor growth and improved survival in an orthotopic TNBC 4T1 model. Combination treatment using both trap genes suppressed immunosuppressive cells such as PD-L1+ cells and M2 macrophages within the tumor [132].

Silybin or silibinin is a flavonolignan of *Silybum marianum*, a medicinal plant that has been used for thousands of years for liver diseases and is known for its antioxidant, anti-inflammatory, and antifibrotic power [156]. Due to its known activity in the treatment of liver fibrosis, Wu et al. investigated the effects of silybin on the eradication of CAFs in the TNBC 4T1 model [133]. Nanoliposomes were fabricated as carriers of silybin to increase its stability and bioavailability. They determined that α-SMA expression was significantly reduced, and immune responses improved through increased IFN-γ, IL-12 and cytotoxic T cells. In vivo, combination treatment with liposomal doxorubicin led to immunogenic tumor apoptosis and prolonged survival. The combination of liposomal silybin with liposomal doxorubicin reduced tumorigenesis and further prolonged the survival of mice [133].

Marimastat is an enzyme inhibitor that is active against gelatinases, collagenases, and metalloproteinases [30]. Hybrid nanoparticles (HNPs) comprising marimastat-loaded thermosensitive liposomes together with the hyaluronic acid-paclitaxel prodrug were designed as a dual-targeting system to deliver agents to the tumor microenvironment and cancerous cells. HNPs released marimastat and the hyaluronic acid-paclitaxel prodrug under mild hyperthermic conditions in the tumor microenvironment [134]. The hyaluronic acid-paclitaxel prodrug impeded the survival of cancerous cells and marimastat decreased TGF-β1, TN-C and α-SMA expression, metalloproteinase activity, and cancer cell migration. Treatment with HNPs in the murine model of TNBC resulted in significant inhibition of tumor growth, metastasis, angiogenesis, and negatively affected some BCAFs [134].

Puerarin is an isoflavone derived from the kudzu root with potential applications in the reduction of blood pressure and myocardial oxygen consumption and in ischemia–reperfusion injury [157]. Puerarin negatively regulated reactive oxygen species production in CAFs [135]. A puerarin nanoemulsion (nano-puerarin) was developed using lecithin and aminoethyl anisamide as the targeting ligand for the sigma receptor, which is abundantly expressed on CAFs and cancerous cells. Additionally, it significantly decreased α-SMA in the murine 4T1 TNBC model compared to the untreated control. This led to a reduction in collagen, BCAF secreted immune suppressive cytokines such as IL-6 and IL-10, and an increase in T cells (CD8+ and CD4+) in the tumor. When nano-puerarin was used in combination with paclitaxel nanoparticles formulated using a nanopolymer system, it increased the efficacy of nano-paclitaxel. Nano-puerarin deactivated desmoplastic TME by decreasing α-SMA positive BCAFs in the TNBC tumor [135].

#### 2.4.3. Polymeric and Hydrogel-Based Nanoparticles

Angiotensin receptors function by binding to the angiotensin II hormone and play crucial roles in the human body, including renal regulation, cell growth, and survival [158]. Angiotensin receptor blockers (ARBs) are antagonists such as valsartan and losartan, which are clinically used to treat high blood pressure [158]. Many ARBs, including valsartan and losartan, are based on a biphenyl tetrazolo structure [159]. In addition to these, ARBs have also been shown to have CAF reprogramming effects and reduce α-SMA+ CAF levels [160]. However, an important challenge is that ARBs can cause hypotension. Hence, novel delivery systems are being demanded to reduce their on-target side effects in cancer application. An ARB nanoconjugate with valsartan attached to polyacetal (1,1,1-Tris(hydroxymethyl)ethane, di(ethylene glycol) divinyl ether and polyethylene glycol) [136]. These nanoconjugates were designed to overcome the hypotension side effect generated by ARBs. Thus, the ARBs were chemically linked with an acid-degradable polymer that is sensitive to the pH of the tumor. These ARB nanoconjugates were examined in murine models, where they accumulated in high concentrations, were active in tumors, and remained inactive in the circulation. This system improved the BCAF-targeting capacity of ARBs and reduced the side effect of lowering the blood pressure. The ARB nanoconjugate significantly decreased α-SMA+ cells (mostly BCAFs, as well as the minute population of other cell types such as pericytes), collagen I expression, and solid stress compared to the unconjugated ARB in the murine TNBC model (4T1). Moreover, the ARB nanoconjugate enhanced the activities of T lymphocytes and immune checkpoint blockers [136].

Losartan is an angiotensin inhibitor that has been approved as a hypertension management medicine [161,162]. It also demonstrated efficacy in reducing cardiac and renal fibrosis [163,164]. In some studies, losartan has inhibited collagen, hyaluronan production, and profibrotic signaling of TGF-β1 in syngeneic TNBC models of mice [160]. There is substantial evidence that losartan can reduce collagen I production and solid stress and negatively impact some BCAFs [160,165]. The peptide (C16-GNNQQNYKD-OH) encapsulated losartan in a hydrogel form. Losartan-loaded nanohydrogels were used to target BCAFs in the murine TNBC model (4T1) [137]. This nanoparticle was delivered locally through intratumoral injection and reduced the expression of α-SMA+ BCAFs and collagen. In addition, it improved the efficacy of chemotherapy (Doxil^®^), suppressed tumor growth, and metastasis [137].

#### 2.4.4. Nano-Ferritins

Ferritin nanocages (10 to 12 nm diameter) are composed of a protein that uses Fe_2_O_3_ in its building blocks, storing iron in its cavity [166]. Holoferritine (H-ferritin) is a type of ferritin that can change iron states (oxidize Fe^2+^ to Fe^3+^) [167]. Sitia et al. targeted BCAFs directly using the H-ferritin nanocage delivery system [138]. The H-ferritin nanocages were conjugated with FAP antibody fragments. Since FAP is expressed on the surface of BCAFs, this enabled the functionalized H-ferritin nanocages to bind specifically to the BCAFs. The conjugated H-ferritin nanocages were loaded with navitoclax, a B-cell lymphoma 2 (BCL-2) inhibitor that acts by inducing apoptosis. Conjugated H-ferritin nanocages loaded with navitoclax (HNav-FAP) induced significantly higher cytotoxicity in BCAFs compared to untargeted H-ferritin nanocages. Furthermore, H-ferritin nanocages conjugated with FAP specifically targeted BCAFs in a syngeneic murine 4T1 TNBC model. As 61% of BCAFs generated from primary culture of murine breast cancer and less than 1% of cancerous cells displayed FAP expression in this study, HNav-FAP induced cytotoxicity in BCAFs, not cancerous cells [138]. Future studies should investigate the sub-groups of BCAFs and how they are impacted by HNav-FAP.

Photodynamic therapy is based on the use of a non-toxic dose of light in cells containing light-sensitive molecules (photosensitizer) [139]. Photodynamic therapy is used as an anticancer therapeutic approach without targeting photosensitizer molecules. Rather, by eliminating cancerous cells by creating reactive oxygen species or damaging the vasculature [168]. Ferritin nanocages with anti-FAP single chain variable fragment antibody and a photosensitizer were utilized to target BCAFs in the syngeneic 4T1 TNBC in vivo model. FAP expressing BCAFs were selectively eradicated due to the localized photoirradiation. Additionally, CXCL12 secretion by BCAFs was reduced, which improved antitumor immunity through increasing CD8+ T-cell infiltration [139]. A similar study was conducted using the FAP-targeted photosensitizer described above to increase the penetration of quantum dots [169]. In this study, quantum dots were used to represent macromolecules or nanoparticles, which often cannot reach the tumor site. FAP-targeted photosensitizer treatment and subsequent irradiation increased the uptake of 10 and 50 nm quantum dots by more than 3-fold. This was due to the suppression of collagen in the extracellular matrix that enhanced the penetration capacity of quantum dots. Both studies demonstrate the ability of BCAF-targeted photosensitizer to reduce collagen, improve antitumor immunity, and drug delivery by nanoparticles [139,169].

## 3. Challenges, Clinical Prospects, and Conclusions

### 3.1. Challenges with Anti-BCAF Agents and Their Corresponding Delivery Systems

The studies of BCAF-targeting therapeutic candidates in this review were rigorously selected based on the ability of the therapeutic candidate to reprogram BCAFs or inhibit BCAF functions in experimental TNBC models. Anti-BCAF agents discussed include small molecules, nucleic acid-based targeting candidates, antibodies, and nanoparticles. Some studies are less reliable than others due to their limitations. Study-specific limitations are evaluated above, and some common limitations are described in this section.

A challenge with anti-BCAF studies is the lack of a standardized approach to validate the presence of BCAFs. Some studies evaluated various markers of BCAFs, including FAP, α-SMA and VIM, and the absence of epithelial markers. Pirfenidone studies used multiple markers to validate that BCAFs were present and impacted by treatment [54,65]. However, some studies did not use multiple markers and, therefore, their evidence was not strong, so more studies are required. For example, the study by the Szebeni laboratory investigating the effects of cisplatin on BCAFs only used one marker, which was the proteolytic activity of FAP [69]. Using one marker, such as α-SMA, to detect the level of BCAFs is not a reliable approach, as myoepithelial cells also express α-SMA and some subpopulations of CAFs have a low expression of α-SMA [68,170]. Hence, it is crucial to have a standard validation method to identify the presence of BCAFs with at least three markers of BCAFs. Another challenge is that there are no immortalized BCAF cell lines, and BCAFs need to be created using diverse experimental designs. Some studies use reliable models, where fibroblasts are co-cultured with TNBC cell lines or have been derived from TNBC xenograft or syngeneic murine models [53,54,65,70,72,82,127]. One study used normal fibroblasts transfected with miR-9 to model BCAFs, while another used CAFs derived from melanomas in a monoculture to determine the impact on BCAFs [81,130]. These models need to be improved by co-cultures or using BCAFs derived from TNBC status verified murine models or patient cancer samples. Another challenge associated with anti-BCAF studies is the lack of having immortalized BCAFs for in vitro studies, which can be addressed by establishing these cell lines to provide a standardized validation approach enabling the detection of at least three key markers.

Some of the therapeutic agents in this review have only been studied in vitro; more in vivo studies are required to progress these anti-BCAF agents into clinical practice. One of such anti-BCAF agents is AMD3100, which demonstrated promising in vitro results as it inhibited the invasion and proliferation of TNBC cells when co-cultured with BCAFs [53]. The advantages of this agent are that it is an approved drug for another disease, it is safe, and it is a CXCR4 antagonist. The CXCR4 antagonist, balixafortide in combination with eribulin, is currently being tested in a phase I clinical trial to only reduce breast cancer metastasis and improve chemotherapy efficacy [171,172]. Therefore, AMD3100 has the potential to be part of a combination strategy in the treatment of TNBC. However, comprehensive in vivo or 3D studies are required for progression to clinical trials.

Some studies used a murine model in which both CAFs and cancer cells were injected into animals [54]. In these studies, the effect of the anti-BCAF agent was more pronounced (due to the additional BCAFs injected). Thus, the impact of these agents on the endogenous levels of BCAFs was not revealed in a way that naturally occurs. Among all, the study of ProAgio by the Liu group used murine models in which only MDA-MB-231 cells were injected [125]. Therefore, this would be a more relevant model, which determined the efficacy of ProAgio as an anti-BCAF agent more accurately.

Despite the promising results of anti-BCAF compounds, some technologies developed to deliver these agents lack specificity for BCAFs. For example, nanoliposomes containing silybin and losartan-loaded hydrogels are examples of systems that can be improved with a BCAF targeting moiety [133,137]. Some of these include the aminoethyl anisamide ligand or the FAP antibody that had been used in nano-puerarin and αFAP-PE38 delivery systems to target sigma receptors and FAP, respectively [129,135].

While it is known that BCAFs have heterogenous subpopulations in TNBC [41], many of the studies did not investigate which subset of BCAFs was impacted by the treatment. Only one study described BCAFs with high and low expression α-SMA in TNBC [68]; however, no further investigation was conducted that allows characterization of the two subsets of BCAFs. Further studies should investigate the sub-groups of BCAFs and the impact of therapeutic candidates on each of the subgroups.

### 3.2. Clinical Prospects

Several clinical trials have been conducted for anti-CAF therapy in cancer [23,173]. However, the majority of studies do not focus on BCAFs in TNBC. For instance, TGF-β receptor inhibitor, galunisertib, was tested in a Phase I clinical trial (NCT02672475) with paclitaxel for a subtype of metastatic TNBC. However, preclinical studies evaluated only the anticancer activity of this compound and not its anti-BCAF potential [174,175]. It is deduced that inhibition of TGF-β is affecting BCAFs’ protumor function, which is mediated by TGF-β [173].

Hedgehog signaling-activated CAFs lead to resistance to drugs and metastasis. Smoothened transmembrane protein (SMO) is part of the Hedgehog signaling pathway. Inhibitors of SMO, vismodegib, and sonedigib reverse the BCAF phenotype of drug resistance. A Phase I clinical trial (EDALINE) reported the use of sonedigib that targets BCAFs in combination with cytotoxic docetaxel [176,177,178]. This trial showed that the treatment was well tolerated (three out of 12 patients with metastatic TNBC found a benefit from this therapy with one patient experiencing complete recovery) [178]. Given that 30% of TNBC patients have an activated Hedgehog pathway, there is a potential that this strategy can treat metastatic TNBC with activated Hedgehog signaling by targeting BCAFs and improving drug sensitivity. This may progress to Phase II clinical trial, which will provide more statistically significant data using a large patient group and additionally provide insight into the effectiveness of the treatment [178]. As BCAFs are emerging therapeutic targets, they are progressively receiving attention. Hence, there is a potential that the anti-BCAF agents discussed in this review will reach clinical trials in the future.

### 3.3. Conclusions

Numerous studies have been conducted to target BCAFs in TNBC through a diverse range of experimental designs. These studies also demonstrated the crucial role of BCAFs in the aggressiveness of TNBC, leading to a poor prognosis. BCAFs contribute from the initiation of cancer to therapy resistance, invasion, and metastasis. Thus, the mechanisms of BCAFs’ activity and strategies for its inhibition have been the focus of the corresponding field of research towards the development of effective anti-TNBC therapies. The therapeutic candidates discussed herein have various structures and targeting potentials. Some were able to reprogram TNBC BCAFs to a quiescent state, and others affected the viability of BCAFs. Furthermore, there were several reports on the improvement of survival in in vivo murine TNBC models. There are several repurposed agents that have been promising candidates with an established safety profile and potential efficacy that can be swiftly translated into clinics. Multitargeted therapeutic candidates are also attractive due to their specificity and multiple mechanisms of action to overcome TNBC progression and drug resistance. Further research on repurposed anti-BCAF drugs and multitargeted therapeutic candidates should be carried out to allow their translation to the clinic. Overall, targeting BCAFs in combination with TNBC cancer cells is a more promising strategy with higher potential to overcome drug resistance, relapse, and metastasis, and it could contribute to reducing the disease burden of this clinically unmet condition.

## Figures and Tables

**Table 1:** BCAF targeting therapeutic candidates (free small molecules and nucleic acid-based agents) in TNBC.

Therapeutic Strategy	Class	Name	Specific Target	Effect
*Free small molecules*	TGF-β inhibitors	Artesunate and dihydroartemisinin [63]	TGF-β	↓ Activation of BCAFs ↓ α-SMA, FAP and fibronectin ↓ TGF-β1 and phosphorylated SMAD3 ↓ Tumor growth and metastasis
		Pirfenidone [54,64,65]	TGF-β	↓ BCAF proliferation and migration ↓ Apoptosis of cancer cells and BCAFs ↓ Fibrosis and fibronectin ↓ Components of extracellular matrix ↓ Mechanical forces ↓ TNF-β secretion and PD-L1 ↓ Tumor growth and metastasis
		+Doxorubicin		
		Tranilast [66] +Doxil	TGF-β and Histamine	↓ Extracellular matrix components ↑ Perfusion, infiltration of T cells ↑ Treatment efficacy and M1 macrophage
		Emodin [67]	TGF-β	↓ Migration of cancer cells ↓ EMT
	Dual targeting agents	Embelin [68]	High α-SMA	↓ α-SMA ↓ Tumor growth
		Cisplatin [69]	FAP	↓ FAP proteolytic activity
		AMD3100 [53]	CXCR4	↓ Cancer cell proliferation ↓ Invasion
		Cabozantinib [70]	MET	↓ Cancer cell growth ↓ Invasion
		Pyrrolo[2,3-d]pyrimidine-based derivatives [71]	LIFR-JAK HDAC	↓ LIFR-JAK signaling ↓ Tumor growth
		DAPT [72]	Notch receptors	↓ CXCL8 ↓ Invasion and migration
*Nucleic acid-based agents*	Small molecules targeting miRNAs	AC1MMYR2 [73,74]	MiR-21	Transformation into normal fibroblasts ↓ α-SMA, FAP-α and EMT ↓ Tumor growth ↓ Tumor growth and lung metastasis
		+Paclitaxel		
		Curcumin [75,76]	MiR-146b-5p	↑ p16 Inactivation and growth arrest of BCAFs ↓ Migration and invasion ↓ Pro-tumorigenic activities of BCAFs
	Mimics and anti-miRNAs	BAMBI [77]	TGF-β/SMAD signaling	↓ BCAF differentiation ↓ Tumor growth and metastasis
		Let-7b mimic [78]	Let-7b	BCAFs inactivation ↓ α-SMA, IL-8, TGF-β1 and CXCL12 ↓ Migration and invasion
		Anti-miR21, -143 and -378e [79]	MiR-21 MiR-143 MiR-378e	↓ Invasiveness ↓ Stemness ↓ EMT
	Aptamers	LNA-i-miR-221 [80]	MiR-221	↓ TNBC and BCAF proliferation ↓ TNBC and BCAF migration
		LNA-9 [81]	MiR-9	↓ Cancer cell migration and invasion ↓ BCAF migration and invasion ↓ TNBC and BCAF cell motility
		Gint4.T [82]	PDGFRβ	↓ BM-MSC differentiation into BCAFs ↓ Pro-metastatic ability

↑ and ↓ indicates the up-regulation and down-regulation, respectively, due to treatment.

**Table 2:** BCAF targeting therapeutic candidates (Antibodies, proteins, and nanoparticles) in TNBC.

Therapeutic Strategy	Class	Name	Specific Target	Effect
*Antibodies and proteins*	Antibodies	Interferon signaling blocking antibodies [124]	Type I and II interferon signaling	↓ BCAF induced chemoresistance
		RG7386 [127]	FAP Death receptor 5 (cancer cells)	Apoptosis of cancer cells ↓ Tumor growth
	Proteins	ProAgio [125]	Integrin αvβ3	Apoptosis of BCAFs ↓ Collagen ↓ BCAF secreted growth factors ↓ Cancer cell proliferation ↓ Tumor growth ↑ Survival of TNBC mice model
	Antibody-drug conjugates	MORAb-202 [128]	Folate receptor alpha (cancer cells)	↓ α-SMA ↓ Tumor growth ↑ Drug efficacy
		αFAP-PE38 [129]	FAP	↓ BCAF activation ↓ BCAF secreted factors ↓ Tumor growth ↓ Tumor growth
		+Paclitaxel		
*Nanoparticles*	Gold nanoparticles	Gold nanoparticles [130]	Integrin αvβ3	↑ Uptake in MDA-MB-231 and CAFs
	Lipid-based nanoparticles	Nanoparticles with TN-C [131]	TN-C	↑ Cytotoxicity ↓ Tumor growth
		Nanoparticles with IL10 and CXCL12 trap genes [132]	Interleukin 10	↓ Tumor growth ↑ Survival of TNBC mice model ↓ Immunosuppressive cells
		Silybin [133]	Nuclear factor-κB	Reprograms BCAFs into fibroblasts ↓ α-SMA ↑ IFN-γ, IL-12 and cytotoxic T cells ↑ Tumor apoptosis and survival
		+liposomal doxorubicin		
		Hybrid nanoparticles [134]	TME (gelatinases, collagenases, and metalloproteinases)+ cancer cells	↓ TGF-β1 expression, ↓ Metalloproteinases activity ↓ Cancer cell survival and migration ↓ BCAFs activation ↓ Tumor growth and metastasis ↓ Angiogenesis
		Nano-puerarin [135]	Sigma receptor	↓ Reactive oxygen species ↓ BCAF activation ↓ BCAF secreted factors ↑ T cells ↓ Tumor growth ↓ Tumor growth
		+ nanopaclitaxel		
	Polymeric and hydrogel-based nanoparticles	ARB nanoconjugates [136]	Angiotensin	Reprograms BCAFs into fibroblasts ↓ α-SMA and collagen I ↓ Solid stress
		Losartan loaded peptide [137]	Angiotensin	↓ TGF-β1 and collagen ↓ BCAFs activation ↑ Drug delivery ↓ Tumor growth ↓ Tumor growth and metastasis
		+ doxorubicin-loaded liposomes		
	Nano-Ferritins	HNav-FAP [138]	FAP	↑ BCAF cell death
		FAP-targeted photosensitizer [139]	FAP	↓ Collagen ↑ Antitumor immunity ↑ Drug delivery

↑ and ↓ indicates the up-regulation and down-regulation, respectively, due to treatment.

## Data Availability

This review article referenced all the articles that were discussed. There were no further data to report.
